# Pneumothorax in COVID-19 disease- incidence and clinical characteristics

**DOI:** 10.1186/s12931-020-01504-y

**Published:** 2020-09-16

**Authors:** Massa Zantah, Eduardo Dominguez Castillo, Ryan Townsend, Fusun Dikengil, Gerard J. Criner

**Affiliations:** Department of Thoracic Medicine and Surgery, Lewis Katz School of Medicine at Temple University, Philadelphia, PA 19140 USA

**Keywords:** Pneumothorax, COVID-19, Spontaneous pneumothorax

## Abstract

**Background:**

Spontaneous pneumothorax is an uncommon complication of COVID-19 viral pneumonia. The exact incidence and risk factors are still unknown. Herein we review the incidence and outcomes of pneumothorax in over 3000 patients admitted to our institution for suspected COVID-19 pneumonia.

**Methods:**

We performed a retrospective review of COVID-19 cases admitted to our hospital. Patients who were diagnosed with a spontaneous pneumothorax were identified to calculate the incidence of this event. Their clinical characteristics were thoroughly documented. Data regarding their clinical outcomes were gathered. Each case was presented as a brief synopsis.

**Results:**

Three thousand three hundred sixty-eight patients were admitted to our institution between March 1st, 2020 and June 8th, 2020 for suspected COVID 19 pneumonia, 902 patients were nasopharyngeal swab positive. Six cases of COVID-19 patients who developed spontaneous pneumothorax were identified (0.66%). Their baseline imaging showed diffuse bilateral ground-glass opacities and consolidations, mostly in the posterior and peripheral lung regions. 4/6 cases were associated with mechanical ventilation. All patients required placement of a chest tube. In all cases, mortality (66.6%) was not directly related to the pneumothorax.

**Conclusion:**

Spontaneous pneumothorax is a rare complication of COVID-19 viral pneumonia and may occur in the absence of mechanical ventilation. Clinicians should be vigilant about the diagnosis and treatment of this complication.

## Introduction

The term spontaneous pneumothorax refers to the presence of air in the pleural space that is not caused by trauma or other obvious precipitating factor (trauma or iatrogenic during a procedure). While primary spontaneous pneumothorax occurs without a clinically apparent lung condition; secondary spontaneous pneumothorax is a complication of preexisting lung disease [1, 2]. To this date, there are only rare mentions of pneumothorax as a complication of COVID-19 viral pneumonia including few case reports [3–6].

The incidence of this complication is still not yet exactly known. In a report by Chen et al. 1% (one patient) had a pneumothorax among other radiographic features [7]. In a study published by Yang and colleagues in 92 deceased COVID-19 patients, one (1.1%) had a pneumothorax and died as a result of it 5 days after the initial presentation [8]. A pneumothorax has also been linked to poor prognosis in patients infected with the acute Middle East respiratory syndrome corona-virus (MERS-CoV) [9].

The proposed mechanism of spontaneous pneumothorax in patients with COVID-19 disease is thought to be related to the structural changes that occur in the lung parenchyma. These include cystic and fibrotic changes leading to alveolar tears. In addition to the increase in intrathoracic pressure resulting from prolonged coughing and/or mechanical ventilation [3, 5, 6, 10, 11].

Herein we review the incidence and outcomes of pneumothorax in over 3000 patients admitted to our institution for suspected COVID-19 pneumonia. We discuss the six cases of patients with COVID-19 pneumonia who developed spontaneous pneumothorax and describe their clinical and radiographic characteristics and outcomes in the context of other cases reported to date.

## Methods

A retrospective review of charts of patients admitted with COVID-19 disease was performed at our tertiary care academic medical center between March 1st and June 8th^th^ 2020. During this time period we treated 902 patients with COVID-19. Their diagnosis was made based on polymerase chain reaction (PCR) testing of nasopharyngeal swab sampling. All patients had a computed tomography (CT) of the chest on admission, in addition to routine daily chest x-ray. The presence or absence of pneumothorax was determined based on review of clinical documentation and chest radiographic imaging. Patients who had a pneumothorax at any time during their clinical course were thoroughly reviewed. Baseline laboratory data including inflammatory markers C-reactive protein (CRP), lactate dehydrogenase (LDH), Ferritin, D-dimer, Interlukin-6, White blood cell count (WBC), absolute lymphocyte and neutrophil counts were documented for each patient.

Respiratory function status was evaluated using the SF ratio. This is defined as the ratio of oxygen saturation as measured by pulse oximetry (SpO_2_) to the fraction of inspired oxygen (FiO_2_) to respiratory rate (SpO_2_/FiO_2_ ratio). The incidence of spontaneous pneumothorax in COVID-19 patients was then calculated. Temple University Hospital Review Board approved the protocol.

## Results

During the study period, we had admitted and treated over 3000 patients with suspected COVID-19 pneumonia, 902 of these patients were nasopharyngeal swab positive for COVID-19. Six patients developed a pneumothorax; the incidence of spontaneous pneumothorax was 0.66%. Characteristics of these patients are summarized in Tables 1 and 2.

**Table 1:** Demographics and clinical characteristics for patients with COVID-19 and pneumothorax

Patient/Sex/Age. yr	Characteristics of Baseline CT Scan	Risk Factor for PTX	Time to onset, days	Size of PTX	Chest Tube	Interval until Resolution, days	Outcomes
1/M/49	Patchy GGOs and consolidations in peripheral distribution bilaterally	None	11	Large	Yes	0.5	Died
2/M/59	Patchy GGOs, consolidations and crazy paving pattern in peripheral distribution bilaterally	Mechanical Ventilation	12		Yes	7–13	Survived
3/F/81	Extensive GGO’s and consolidative changes bilaterally with predominant consolidation in the RUL	None	9	Moderate	Yes	3–14	Died
4/F/45	Extensive bilateral consolidative and ground glass opacities	Mechanical Ventilation	1	Small	Yes	1	Survived
5/F/47	Bilateral consolidations and ground glass opacities.	Mechanical Ventilation	12	Small	Yes	1	Died
6/F/76	Bilateral basilar consolidations	Mechanical ventilation ILD	21	Large	Yes	1	Died

**Table 2:** Baseline laboratory and respiratory function data for patients with COVID-19 and pneumothorax

Case	1	2	3	4	5	6
Baseline Laboratory/ Inflammatory Markers Data						
WBC (K/mm^3^)	8.1	8.3	4.3	4.5	11.5	7.4
Absolute Lymphocyte Count (K/mm^3^)	0.6	0.8	0.5	0.6	0.5	0.7
Absolute Neutrophil Count (K/mm^3^)	4.5	7.0	3.1	3.4	7.8	6.5
CRP (mg/L)	13.1	13.6	11.3	16.2	8.7	16.5
LDH (U/L)	331	472	148	200	481	386
D- Dimer (ng/ml)	695	788	662	993	1164	6947
Ferritin (ng/ml)	676	775	419	268	834	446
IL-6 (pg/ml)	80.37	70.39	< 1.4	6.69	11.95	–
Baseline Respiratory Function						
SF ratio	215	156	218	133	92	155

### Review of the cases

#### Case 1

49-year-old male with morbid obesity, (BMI 47.2), coronary artery disease and heart failure, presented with a one-week history of fever, nonproductive cough, anosmia and shortness of breath. The patient never smoked and had no underlying lung disease. He required oxygen via nasal cannula (SF ratio 215). His chest x-ray (CXR) showed infiltrates in peripheral midlung zones and bases bilaterally. A CT scan of the chest showed patchy ground glass opacities and consolidations in a peripheral distribution in both lungs. His nasopharyngeal swab for SARS CoV-2 was positive. His laboratory findings were significant for lymphopenia and elevated inflammatory markers. He was admitted to the hospital and received antibiotics, corticosteroids, convalescent plasma and off label Tocilizumab. A week later, the patient developed worsening hypoxia (PF ratio of 65) and was placed on a high-flow nasal cannula (HFNC). A CT angiogram was done to rule out possible PE and demonstrated a large left sided pneumothorax. A chest tube was placed using an open surgical approach. His pneumothorax resolved within 12 h. His hospital course was complicated by worsening hypoxia due to COVID-19 pneumonia, necessitating initiating invasive mechanical ventilation and placement of venous-venous extracorporeal membrane oxygenation (ECMO). Patient developed multiorgan failure and died despite providing resuscitative measures.

#### Case 2

59-year-old male with obesity (BMI 32.77) presented with 10-days symptoms of fatigue, fevers, cough and shortness of breath. He was febrile and hypoxic needing oxygen via HFNC on admission (SF ratio 156). His labs were significant for lymphopenia and elevated inflammatory markers. A CXR showed multifocal bilateral airspace opacities. A CT scan of the chest showed areas of crazy paving, diffuse large areas of mixed consolidative and ground glass opacities. His nasopharyngeal swab for SARS-CoV-2 was positive. He was started on antibiotics and corticosteroids. In addition, the patient later received convalescent plasma and off label Tocilizumab. He was intubated 2 days later given persistent hypoxia and increased work of breathing while on HFNC (PF ratio 61). Ten days after intubation, the decision was made to proceed with percutaneous tracheostomy given prediction of prolonged intubation. Prior to the procedure, the ventilator settings were respiratory rate (RR) 16 bpm, tidal volume (TV) 450 ml, FiO_2_ 60% and positive end-expiratory pressure (PEEP) 10 cm H_2_O. His peak inspiratory pressure (PIP) was 26 and plateau pressure 22 cm H_2_O. During the procedure, and soon after placement of the endotracheal tube, the patient had gradual oxygen desaturation and became hemodynamically unstable. High peak pressures were observed on the ventilator. Bedside ultrasound revealed absence of lung sliding bilaterally. Given concern for tension pneumothorax, bilateral chest tubes were placed via surgical approach. The patient’s hemodynamics and oxygenation improved. An endotracheal tube (ETT) was then successfully placed. A postprocedural CXR showed subcutaneous emphysema but no residual pneumothorax. He was weaned off the ventilator a week later. Eventually his chest tubes were removed, and he was discharged to an acute rehabilitation facility.

#### Case 3

81-year-old female with prior history of hypertension and a stroke presented with progressive fatigue, loss of appetite and diarrhea. Her labs were significant for lymphopenia and mildly elevated inflammatory markers. She was diagnosed with COVID-19 disease based on PCR nasopharyngeal swab 5 days prior to her presentation. A CT scan of the chest showed diffuse bilateral patchy ground glass opacities and consolidations in the peripheral, posterior lung regions. She initially required 4 l of oxygen via nasal cannula (SF ratio of 218). She was started on corticosteroids, antibiotics, and received Remdesivir (within a clinical trial). Soon after her admission, she became more hypoxemic needing oxygen via HFNC. Her chest imaging revealed increased bilateral consolidations. Four days after, she was found to have a small right basilar pneumothorax that enlarged within 2 days. A decision was made to place a chest tube under CT guidance. Three days after resolution of the pneumothorax, the chest tube was removed. Patient’s oxygen requirements again increased a few days later, and a repeat chest x-ray revealed a new large left sided pneumothorax and a recurrent small right sided pneumothorax. A chest tube was placed on the left with resolution of the pneumothorax. Unfortunately, on the following days the patient’s clinical status continued to worsen and she expired after comfort measures were taken.

#### Case 4

45-year-old female with no significant history presented with shortness of breath and cough. She was febrile and hypoxic on admission with SpO_2_ of 80% requiring 15 l oxygen via nasal cannula (SF ratio 133). Lymphopenia and elevated inflammatory markers were noted on initial blood work. Her COVID-19 PCR swab was positive. She underwent a CT scan of the chest which demonstrated extensive bilateral consolidative and ground glass opacities. Her oxygen requirements soon increased up to 50LPM and 100% FiO_2_ via HFNC. She was intubated and initial ventilator settings were RR 18 bpm, TV 450 ml, FiO_2_ 100% and PEEP of 10 cm H_2_O with a resulting PF ratio of 148. Post-intubation CXR revealed right mainstem bronchial intubation and an atelectasis of the left lung. The ETT was then retracted to a proper position and a repeat imaging revealed a small left-sided apical pneumothorax. A chest tube was placed using a thoracostomy approach that resulted in resolution of the pneumothorax. The following day, no further air leak was observed. She was treated with corticosteroids, antibiotics, convalescent plasma and off label Tocilizumab. The chest tube was removed on day 14 after the patient’s recovery and extubation. The patient was eventually weaned off oxygen. She is currently undergoing rehabilitation in the hospital.

#### Case 5

47-year-old woman with a history of meningioma, diabetes, hypothyroidism and obesity (BMI 33.6) presented with a 2-weeks history of dry cough, fevers and fatigue. Upon presentation she was febrile, tachypneic and hypoxic requiring oxygen via HFNC (SF ratio 92). Her blood work was concerning for lymphopenia and elevated inflammatory markers. A CT angiogram of the chest showed a saddle pulmonary embolism, bilateral consolidations and ground glass opacities. She was started on broad-spectrum antibiotics, corticosteroids, anticoagulation with intravenous continuous Heparin and later received off label Tocilizumab. Three days later, the patient was noted to be more hypoxic with increased work of breathing requiring mechanical ventilation. The ventilator settings were RR 18 bpm, TV 400, FiO_2_ 60% and PEEP 10 cm H_2_O. Her PIP and plateau pressure were 27 and 23 cm H_2_O respectively. Twelve days into her hospitalization, the patient was noted to be more hypoxic requiring increasing FiO_2_ on the ventilator. Her CXR revealed bilateral pneumothoraces, with the right being larger than the left. A right sided chest tube was placed using a surgical approach. Her pneumothorax resolved within 2 days. Her hospital course was complicated by disseminated intravascular coagulation (DIC), renal failure and ventilator associated pneumonia. She later developed multiorgan failure. She died as a result of cardiac arrest due to worsening acute respiratory distress syndrome and septic shock.

#### Case 6

76-year-old female with a history of pulmonary sarcoidosis, obesity (BMI 43.15) and hypertension who initially presented progressive muscle weakness concerning for sensorimotor polyneuropathy and myopathy. Due to worsening hypercapnia and somnolence in the settings of muscle weakness, she required mechanical ventilation followed by tracheostomy tube placement. While in the hospital she became persistently febrile and hypoxic (SF ratio 155 and PF ratio 143). Her chest imaging showed bilateral consolidations, more pronounced at the bases, in addition to atelectatic changes on the left. She tested positive for COVID-19. Her hospital course was complicated by bilateral lower extremity deep venous thrombosis, ventilator associated pneumonia with pseudomonas, bacteremia, fungemia and septic shock. After a long hospital course, and despite remaining on the same ventilator settings RR 14 bpm, TV 450 ml, FiO_2_ 100%, PEEP of 12.5 cm H_2_O with observed PIP 20 and plateau pressure 16 cm H_2_O, she developed a large right sided pneumothorax and a chest tube was placed via surgical approach that resulted in lung expansion. Unfortunately, the following day she had a cardiac arrest. She died despite providing resuscitative measures.

## Discussion

An underlying pulmonary disease is the primary risk factor for the development of secondary spontaneous pneumothorax. These include chronic obstructive pulmonary disease (COPD) with emphysema, cystic fibrosis, tuberculosis, lung cancer, HIV associated Pneumocystis jiroveci pneumonia (PJP), and other pulmonary cystic lung diseases [1, 2, 12].

Patients with COVID-19 infection can develop severe pneumonia leading to acute respiratory distress syndrome (ARDS). Their disease is characterized radiographically by ground glass opacities, evolving into consolidative changes and in late stages of the disease, fibrotic changes [8, 13]. Similar changes including severe lung injury and diffuse alveolar damage were thought to contribute to the mechanism of spontaneous pneumothorax complicating severe acute respiratory syndrome (SARS) [14]. These changes, in addition to possible overdistention of the alveoli by using mechanical ventilation may put patients at risk for developing pneumothorax. Table 3 summarizes the literature report that has been published on pneumothorax in COVID-19 patients.

**Table 3:** Summary of literature report on pneumothorax in COVID-19 patients. Data are presented to compare clinical and radiographic characteristics and clinical outcomes

	Age yr/Gender	CT characteristics	Complication	Risk Factors	Time to onset, days	Chest tube	Time to resolution (days)	Outcomes
Zhou et al. [3]	38/M	Bilateral GGOs and consolidations in the lower lobes	Pneumomediastinum	None	11		14	Survived
Wang et al. [5]	36/F	Bilateral Patchy GGOs and consolidations	Pneumomediastinum	NIV	Day 0–12 days after onset of symptoms			Died due to ARDS
Sun et al. [6]	38/M	Patchy peripheral GGOs. Progression to consolidations and bullae	Mediastinal Emphysema, Giant Bulla, Pneumothorax	NIV	Pneumomediastinum 7 days Bullae 21 days PNX 30 days	None		
Aiolfi et al. [4]	56/M 70/M	Bilateral peripheral GGOs	Pneumothorax	Invasive Mechanical Ventilation Preexisting emphysema	2 and 5 days after intubation	Yes		Thoracotomy and bleb resection were performed for persistent pneumothorax
Liu et al. [11]	38/M	Bilateral Patchy GGOs and consolidations, progression to cystic formation	Pneumothorax	None	26	None	5	Survived
Wang et al. Wang et al. [10]	62/M	Bilateral areas of GGOs in the peripheral areas	Pneumomediastinum Pneumothorax Subcutaneous emphysema	None	20	None	16	Survived

In this case series, we identified 6 out of 902 patients with COVID-19 pneumonia who developed spontaneous pneumothorax. The incidence (0.66%) is lower than what has been recently reported in the literature [7, 8].

Mechanical ventilation appears to be a predominant risk factor for development of pneumothorax with COVID-19 pneumonia. Aiodfi and colleagues reported two cases of COVID-19 pneumonia patients who developed persistent pneumothorax while on mechanical ventilation [4]. However, Wang et al. reported a case of a patient who developed spontaneous pneumothorax, pneumomediastinum and subcutaneous emphysema in a patient who was not on any mechanical ventilation [10].Similarly, Zhou et al. reported a case of spontaneous pneumomediastinum in a patient with COVID-19 who was spontaneously breathing [3].While the incidence of pneumothorax in mechanically ventilated patients is high, it is believed to be even higher in those with ARDS, ranging between 14 to 87%. It correlates directly with the severity and duration of ARDS, barotrauma and volutrauma caused by mechanical ventilation. This particularly happens in cases of high peak inspiratory pressures (PIP) (greater than 40 to 50 cm **H**_**2**_**O**), high positive end-expiratory pressure (PEEP), high tidal volumes and minute ventilations [15–17]. Furthermore, ARDS represents a heterogeneous group of a condition in which a conglomerate of relatively healthy and diseased alveoli mix. Commonly, the dependent areas of the lung tend to consolidate due to interstitial edema and represent regions of decreased lung compliance. During lung recruitment maneuvers while managing ARDS, overdistention of “normal” non-dependent lung regions with relatively higher compliance and less airway resistance occurs. These alveoli then can rupture due to disproportionate distribution of volume and pressure from the ventilator causing increase shear forces. In our cases, 4/6 (66%) were on mechanical ventilation. However, these patients had higher lung compliance, indicated by relatively lower peak inspiratory and plateau pressures. In fact, a recent report on patients with COVID-19 pneumonia hypothesized that a subset of patients may exhibit near normal lung compliance [18].

Gattinoni et al. found that the incidence of pneumothorax is higher in patients with ARDS who are on mechanical ventilation for a long duration of time (87% vs. 30% in those with > 2 weeks of mechanical ventilation vs. < 1 week). In addition, patients with bullae and low lung compliance had higher rates of pneumothorax, which are findings that were not applicable to our patients [18]. Regardless, it is essential that low-volume and pressure-limited strategies are utilized as lung protective strategies to minimize barotrauma and volutrauma. We believe that these measures can be beneficial even in those patients with high lung compliance and severe hypoxemia. Further studies evaluating this specific population and the effects of protective mechanical ventilation strategies are necessary to help confirm these hypotheses. It is also important to recognize that to this date, there are no clear guidelines on the timing and settings of mechanical ventilation in patients with COVID-19 pneumonia. High-flow oxygen therapy may be a safer alternative to avoid the potential complications of mechanical ventilation in these patients.

Another possible triggering factor is prolonged coughing, which is a common symptom of COVID-19 disease [7, 19, 20]. 4/6 patients reported cough as a predominant symptom in their presentation. Cough may enhance leakage of air out of the alveoli by causing sudden lengthening and shortening of the pulmonic vessels and associated bronchi during respiration and further moving the “train of bubbles” along the vascular sheaths [21].

**Fig. 1:**
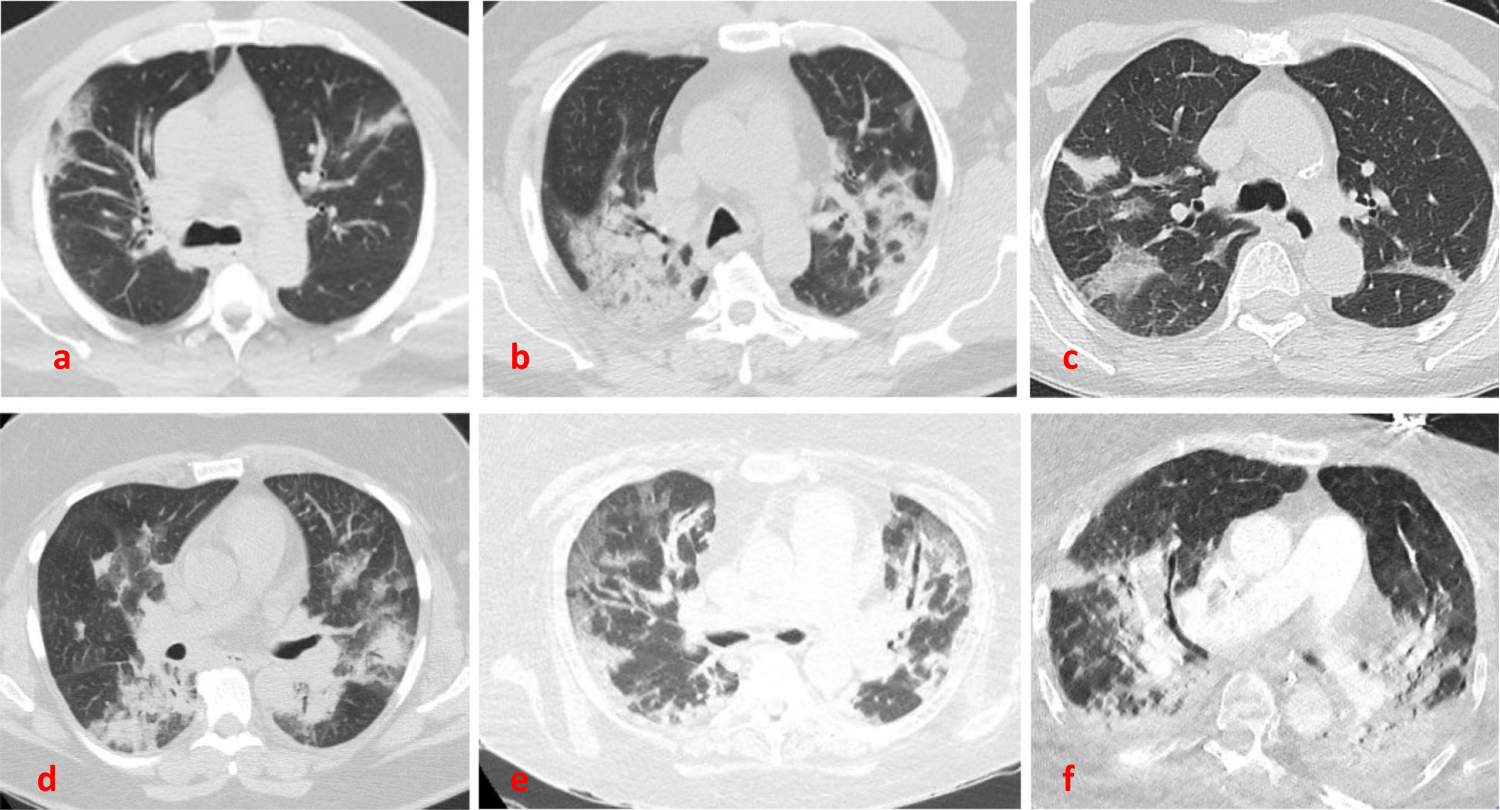
Baseline CT scan of the chest. Areas of GGOs, consolidations and crazy paving seen commonly bilaterally, more predominant in the posterior and peripheral lung regions. From top left: **a** Case-1. **b** Case −2. **c** Case −3. **d** Case-4. **e** Case-5. **f** Case 6

**Fig. 2:**
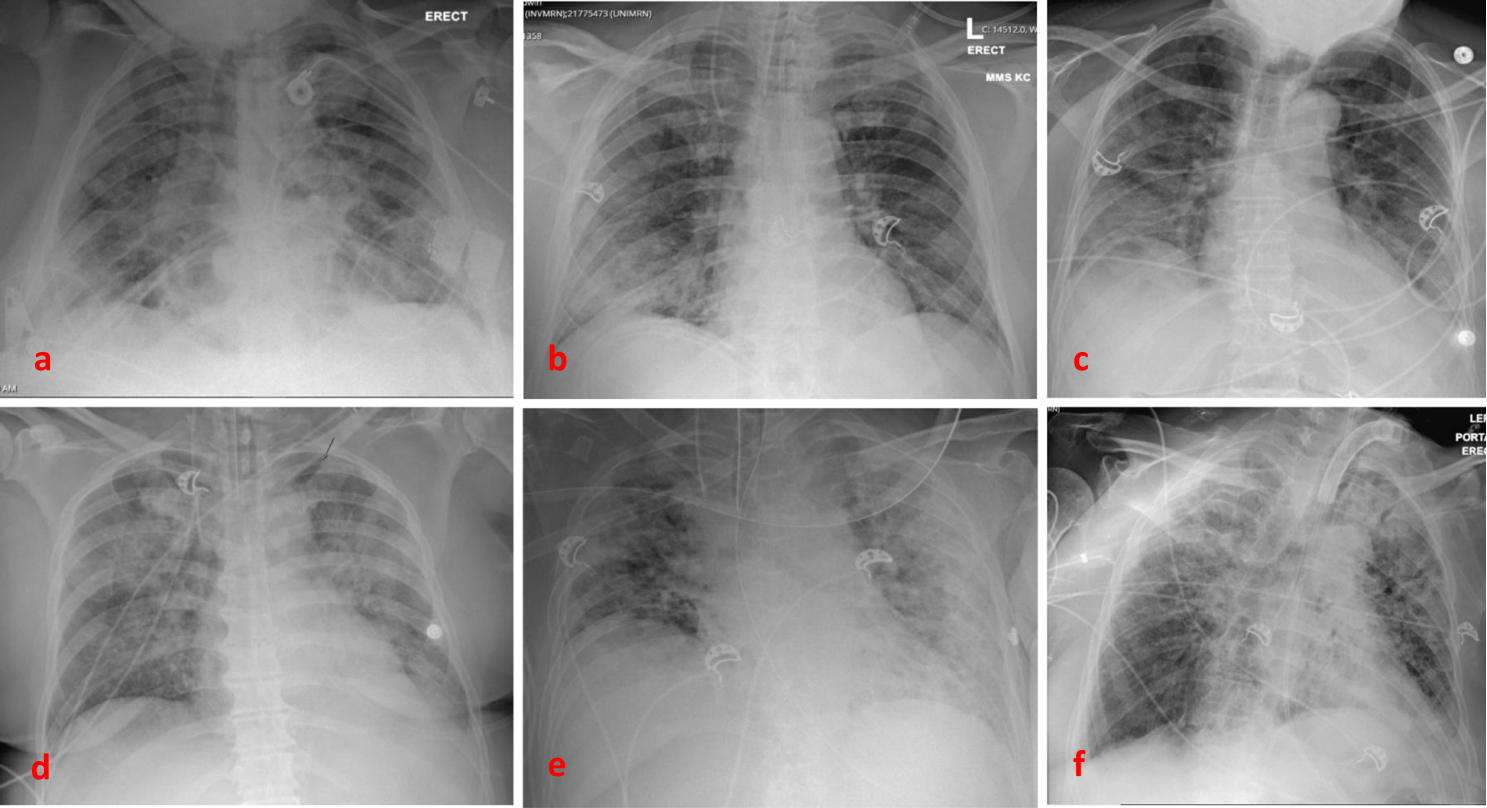
Interim chest radiographs for each patient prior to developing pneumothorax. From top left: **a** Case-1. **b** Case − 2. **c** Case − 3. **d** Case-4. **e** Case-5. **f** Case 6

**Fig. 3:**
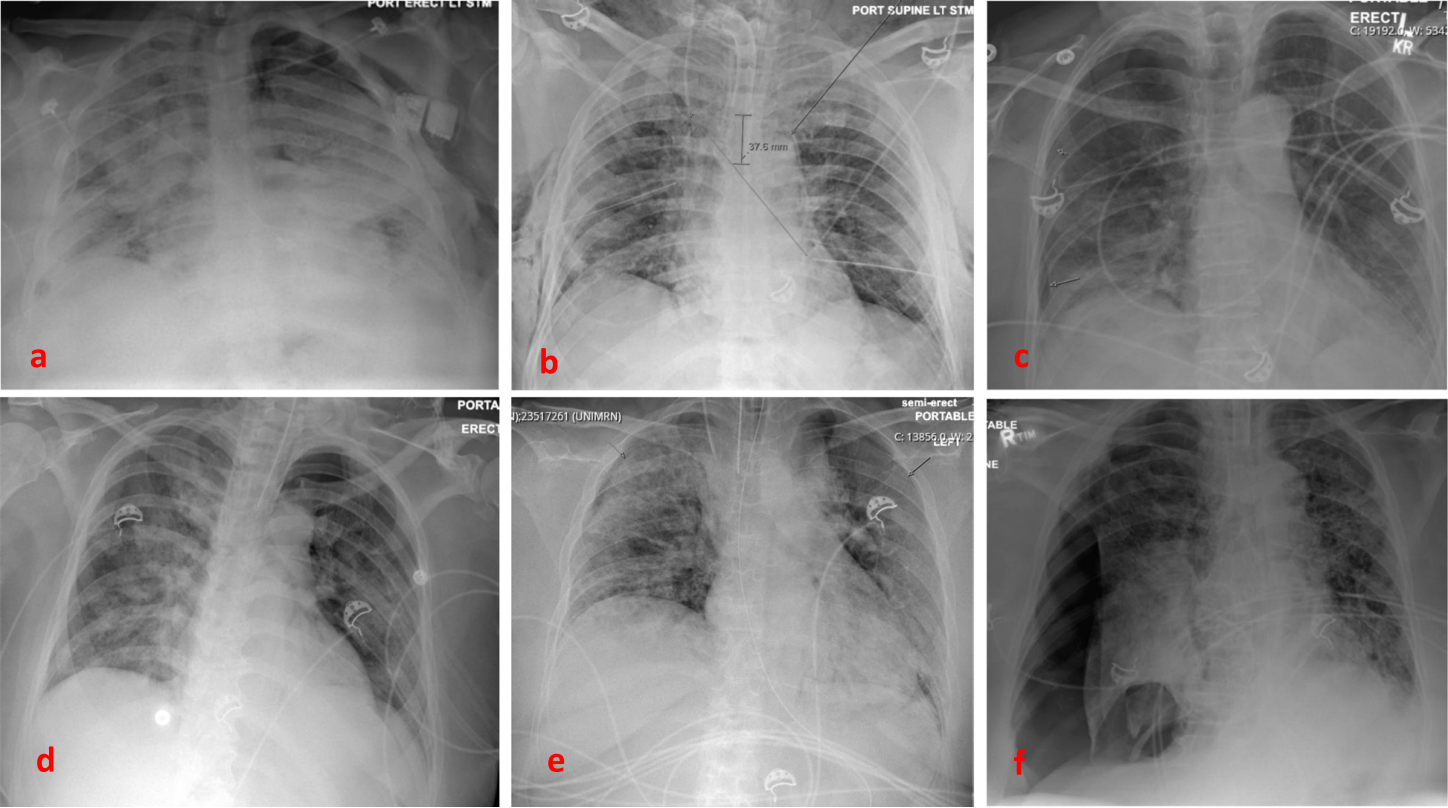
Chest radiograph for each patient revealing the pneumothorax. From top left: **a** Case-1 Large, left. **b** Case − 2 after pneumothoraces developed and chest tubes were placed. Residual right sided subcutaneous emphysema. **c** Case − 3. Moderate, right. **d** Case-4. Small, left apical **e** Case-5. Small, bilateral **f** Case- 6. Large, right

Baseline imaging may be predictive for patients who may develop pneumothorax. A recently published article reported two cases of patients with cystic lung lesions related to COVID-19 disease, one of which developed pneumothorax [11]. This resembles cases of spontaneous pneumothorax in HIV patients and PJP pneumonia [22]. Another case report described a patient with no baseline lung disease who developed a giant bulla as a result of COVID-19 infection, that ended up rupturing and causing pneumothorax [6]. Our patients had different degrees of ground glass opacities, areas of consolidation, crazy paving and atelectasis, consistent with what has been reported in the literature [13, 23]. In our institution, each patient with COVID-19 disease undergoes a CT scan of the chest on admission (Fig. 1), as well as a chest radiograph on a daily basis to help guide clinical decisions and medical management. We were able to capture the radiographic progression of the parenchymal findings on most of the patients prior to development of the pneumothorax (Figs. 2 and 3).

An important finding of our study was the presence of lymphopenia and elevated inflammatory markers including CRP, LDH, Ferritin, D-dimer, and IL-6 levels in almost all patients who developed spontaneous pneumothorax (Table 2). This is consistent with recently published studies, that have examined the possible mechanisms of COVID-19- induced lung injury. Cytokine storm has been thought to play a role in disease pathophysiology. This form of hyperactive and dysregulated immune response may lead to hyperinflammatory form of ARDS and is associated with critical illness and increased mortality [24–26]. Furthermore, thrombosis and microangiopathy was observed in lung tissue obtained from patients with ARDS who died from COVID-19 viral infection. This is thought to play a role in lung injury; however, further studies are needed to examine the implication of these findings [27]. In our institution, all patients with COVID-19 infection were placed on prophylactic or therapeutic anticoagulation, unless contraindicated. The choice and dose of the anticoagulation were based on a multidisciplinary discussion that included the hematology specialists. The decision took into account the inflammatory marker levels, specifically D-dimer values.

Finally, we included one case of what appears to be spontaneous bilateral pneumothorax during percutaneous tracheostomy tube placement. The etiology of this complication is stipulated to be an injury to the anterior or posterior tracheal wall damaging the pleural cavity [28, 29]. Our patient did not have any visible tracheal tears. We believe the most likely mechanism of his bilateral pneumothorax was the combination of positive pressure ventilation, mucous impaction and the inflamed lung parenchyma related to COVID-19 disease. Heterogeneous overdistention of the alveoli due to mucus impaction and/or consolidative phase of COVID-19 viral pneumonia can increase the risk of spontaneous pneumothorax. Furthermore, the overdistension may occur in alveoli with inflammation (areas with GGOs). Although this is a rare complication during tracheostomy placement, proceduralists should be aware of pneumothorax being a potential higher risk in patients with COVID-19 with diffuse parenchymal disease, and a special care must be taken when handling airways of these patients.

## Conclusion

Spontaneous pneumothorax is a rare complication of COVID-19 viral pneumonia. It may occur at any time during the course of the disease. Patients with baseline ground-glass opacities and consolidations and those who are mechanically ventilated appear to be at high risk. Clinicians should be vigilant about the diagnosis and treatment of this complication.

## Data Availability

The datasets used and/or analyzed during the current study are available from the corresponding author on reasonable request.

## Abbreviations

MERS-CoV: Acute Middle East Respiratory Syndrome Corona-Virus
PCR: Polymerase chain reaction
CT: Computed Tomography
BMI: Body Mass Index
CXR: Chest x-ray
HFNC: High-Flow Nasal Cannula
PIP: Peak inspiratory pressure
PEEP: Positive end-expiratory pressure
ARDS: Acute respiratory distress syndrome
ECMO: Extra-corporeal membranous oxygenation
ETT: Endotracheal tube
RR: Respiratory rate
TV: Tidal volume
FiO_2_: Fraction of inspired oxygen
PTX: Pneumothorax
SARS: Severe acute respiratory syndrome
DIC: Disseminated intravascular coagulation
HIV: Human immunodeficiency syndrome
PJP: Pneumocystis jiroveci pneumonia
LDH: Lactate dehydrogenase
CRP: C-reactive protein
IL-6: Interleukin-6
GGOs: Ground glass opacities
SF ratio: SpO_2_/FiO_2_ ratio
PF ratio: PaO_2_ / FiO_2_ ratio
NIV: Noninvasive ventilation
